# A socially just recovery from the COVID-19 pandemic: a call for action on the social determinants of urban health inequalities

**DOI:** 10.1177/0141076820948817

**Published:** 2020-09-11

**Authors:** Katarina Hoernke

**Affiliations:** Institute for Global Health, 4919 University College London, London WC1E 6BT, UK

## Introduction

From bustling business districts to vibrant cultural life, many of the things that make life in cities advantageous can be attributed to their ability to bring people together. Diverse social networks, which promote productivity and innovation, can, however, act as a vector for disease transmission during a pandemic. The social distancing and lockdown measures implemented during the COVID-19 pandemic have brought fast-paced city life to a standstill, giving citizens pause to recognise the necessity of adequate living conditions, the value of access to healthcare and the privilege of digital technology. Many of these response efforts have left the most socioeconomically disadvantaged at greater risk of catching and dying from COVID-19. They have exacerbated existing health inequalities and given rise to new ones. Many are calling for a socially just recovery, including 5 million health professionals representing over 50 countries in their open letter to the United Nations calling for ethical global leadership.^1^ The need for action on health inequalities was evident before the pandemic but this crisis provides an unprecedented opportunity for change and cities can be a focal point for this.

While each city will require a unique recovery strategy from the pandemic, this commentary calls for a health equity approach to be universally adopted. Grounded in the Social Determinants of Health framework,^2^ this commentary suggests three ways cities could change as a result of the COVID-19 pandemic in the housing, healthcare and technology sectors. Public and urban health professionals could engage multi-level stakeholders from civil society to governments at local and national levels to improve daily living conditions, promote access to healthcare and harness the power of digital technologies to address urban health inequalities.

## Social determinants of urban health in the COVID-19 pandemic

Individuals from the most deprived areas of the UK are dying from COVID-19 at twice the rate as those from the least deprived areas, particularly in urban settings such as London.^3^ Across the world we are seeing similar patterns of inequalities. A recent US report found that 70% of people who died of COVID-19 in Chicago were black, despite accounting for only 30% of the population.^4^ Nearly all these individuals were living in five of Chicago’s most deprived neighbourhoods. In India, 22% of the population living in urban informal settlements are shouldering the majority of COVID-19 cases.^5^ These trends can be explained through the Social Determinants of Health framework,^2^ which describes how the socioeconomically disadvantaged suffer poorer health outcomes due to a lack of access to adequate housing, health and social care, as well as education and employment opportunities. This effect is particularly amplified within cities, where rapid urbanisation has often left governments unable to provide the conditions necessary for a healthy life.

During the COVID-19 pandemic, overcrowded living conditions without sanitation facilities have made hand hygiene and physical distancing nearly impossible.^6^ Overrepresented in the informal and essential work sector, many of the poorest urban residents are unable work from home nor afford to take indefinite unpaid leave. Risking their health daily, many are faced with the choice between their health and essential income. Compounding these inequalities, those living in socioeconomic deprivation are at greater risk of dying from COVID-19 as they are more likely to be exposed to smoke and air pollution, suffer from underlying co-morbidities and face barriers in access to adequate healthcare.^7^

The recovery strategies of every city, within and between countries, will be different. One similarity they must share, however, is that health equity is placed at the centre. The Social Determinants of Health framework,^2^ offers guiding principles for a socially just recovery from the COVID-19 pandemic by addressing the underlying determinants of health. Three key determinants which cities could target are living conditions, healthcare and technology.

## Living conditions

Many cities across the world are largely failing to address the inadequate living conditions of their most deprived residents. The one billion people living in urban informal settlements across the world are bearing much of the COVID-19 disease burden due to their already neglected, overcrowded living conditions lacking basic water, sanitation and hygiene (WASH) facilities.^6^ The inadequate living conditions of the urban deprived must be urgently improved. Community mobilisation initiatives engaging citizens to address their own needs through local governance models in partnership with multi-level stakeholders have shown promising results. Take, for example, the project being piloted in informal settlements in Mumbai, Cairo and Kinshasa that maps living density and WASH facilities to improve resource allocation to the most deprived areas in response to the COVID-19 pandemic.^8^ Such initiatives can give agency to the voices of the marginalised which often go unheard as well as benefit from rich local knowledge, which easily goes ignored in top-down response efforts. Longer-term investments could prioritise adequate, affordable housing which does not displace residents. Cities should aim to adopt progressive housing policies that ensure adequate living conditions through equitable rent and housing laws, as well as provide the right to secure tenure.

## Healthcare

The urban poor have been overrepresented in the millions of people facing barriers to healthcare during the COVID-19 pandemic.^7^ Recently unemployed workers relying on employer-based health insurance may not have access to adequate healthcare and face being pushed further into poverty by high out-of-pocket healthcare expenditures, experiencing delays in both diagnosis and treatment of COVID-19.^9^ Even in countries with national health coverage, such as the UK, social gradients of health persist through barriers in access to healthcare. Exempting treatment for COVID-19 from the National Health Service (NHS) charging scheme without removing charges for underlying conditions, does little to reduce barriers in access to healthcare for refugees and migrants in the UK.^10^ The US Families First Coronavirus Response Act provides some free testing, but falls short by not including treatment.^11^ These piecemeal responses are insufficient and undermine the health needs of the most disadvantaged. This pandemic has demonstrated the importance of adequate access and provision of healthcare. Universal health coverage will likely be high on every country’s agenda and cities will play a vital role in reaching this goal. While the structure and financing of healthcare systems occur mainly at the national level, urban planners and public health professionals can play a vital role in ensuring the provision of equitable healthcare delivery to all citizens. Compared to their rural counterparts, urban healthcare facilities are greater in size and resources. These facilities can support integrated primary and secondary healthcare systems and promote strong partnerships between the health and social care sectors. The most disadvantaged can be safeguarded in cities through healthcare service times catered to the work hours of essential workers, as well as community outreach services provided to urban areas geographically distant from healthcare facilities.^12^

## Technology

Increasing digitisation of urban life could perpetuate or break the cycle of health inequalities. Access to digital technology has transitioned urban life to online platforms, allowing many to socialise online and work from home. Importantly, it has also allowed many to access health information and healthcare virtually. From surveillance technologies to enhanced data sharing, many digital public health technologies which were deemed unacceptable to citizens only a few months ago are now being piloted in cities across the world to reduce the spread of COVID-19.^13^ These privileges, however, are not available to all. Many of the socioeconomically disadvantaged suffer from a digital divide with multiple barriers in access to digital technologies due to high costs and unreliable Internet, as well as low digital literacy and technological support.^14^ Inequalities in access to technology could be addressed by providing reliable, ubiquitous Internet and raising digital literacy rates through technical outreach support.^7^ A digital health equity framework approach,^15^ which engages marginalised communities at all levels of conception, design and implementation, could help ensure that the urban poor are not excluded from digital public health initiatives. Finally, these digital public health technologies ought to be driven by public, not corporate, interest.

## Conclusion

The COVID-19 pandemic, as well as the subsequent response measures, have laid bare the stark health inequalities built into the physical infrastructure of our cities and woven into the social fabric of the communities that inhabit them. The rapid, unsustainable urbanisation occurring in cities across the world will likely increase demand for essential urban services, such as housing and healthcare. Health inequalities are a choice. This commentary calls upon urban and public health specialists to work with multi-level stakeholders to address inadequate living conditions for the most socioeconomically disadvantaged, promote equitable provision of healthcare and ensure health equity is central to the increasing digitisation of urban life. With all eyes currently on public health, let us seize this opportunity to build back not just health, but health equity, into cities.

## Declarations

**Ethics approval:** Not applicable.

**Guarantor:** KH.

**Contributorship:** Sole authorship.

**Acknowledgements:** None.

**Provenance:** Not commissioned; peer-reviewed by Johanna Lynch.
